# Osteochondritis Dissecans of the Elbow in Overhead Athletes: A Comprehensive Narrative Review

**DOI:** 10.3390/diagnostics14090916

**Published:** 2024-04-28

**Authors:** Andro Matković, Thomas Ferenc, Damjan Dimnjaković, Nikolina Jurjević, Vinko Vidjak, Branka R. Matković

**Affiliations:** 1 Department of Diagnostic and Interventional Radiology, Merkur University Hospital, 10000 Zagreb, Croatia; andro.matkovic@gmail.com (A.M.);; 2 Department of Orthopedic Surgery, University Hospital Centre Zagreb, 10000 Zagreb, Croatia; 3 School of Medicine, University of Zagreb, 10000 Zagreb, Croatia; 4 Faculty of Kinesiology, University of Zagreb, 10000 Zagreb, Croatia

**Keywords:** osteochondritis dissecans, elbow, overhead athletes

## Abstract

Osteochondritis dissecans (OCD) of the elbow mainly occurs in overhead athletes (OHAs). This narrative review aimed to comprehensively analyze the epidemiological data, etiological factors, clinical and imaging features, treatment options, and outcomes of OHAs with the diagnosis of elbow OCD. A literature search was performed in PubMed/MEDLINE, Scopus, and Web of Science. Individuals with elbow OCD were usually 10–17 years of age with incidence and prevalence varying between studies, depending on the sport activity of the patients. The etiology of OCD lesions is multifactorial, and the main causes are believed to be repetitive trauma, the biomechanical disproportion of the articular surfaces, poor capitellar vascular supply, and inflammatory and genetic factors. Athletes usually presented with elbow pain and mechanical symptoms. The mainstay for the diagnosis of elbow OCD is MRI. The treatment of elbow OCD lesions should be conservative in cases of stable lesions, while various types of surgical treatment are suggested in unstable lesions, depending mainly on the size and localization of the lesion. The awareness of medical practitioners and the timely diagnosis of OCD lesions in OHAs are key to favorable outcomes.

## 1. Introduction

Prior to the COVID-19 pandemic, 60 million children (ages 6–18) annually participated in organized sports, with a tendency for earlier sports involvement [1]. Engaging in sporting activities at an early age has numerous health benefits; however, it also increases the risk of injuries [2]. Different sports are associated with different types of injuries, and influential factors are often age, gender, and competitive or recreational participation [2]. Sports with overhead motions (e.g., baseball, gymnastics, tennis, basketball, handball, water polo, etc.) are known for upper extremity joint injuries, especially the shoulder and elbow [1,2,3,4,5]. One study reported that 11% of sport-related injuries are associated with these two joints, and among overhead athletes (OHAs), this rate increased to over 30% [6]. Baseball is one of the most common overhead sports with an increased risk of developing OCD. Over 80% of prior healthy baseball players reported arm pain during or after specific motions, such as throwing or pitching [7].

Osteochondritis dissecans (OCD) is an acquired, focal lesion of the subchondral bone and often the articular cartilage with variations in the degree of resorption, fragmentation, and bone sclerosis, however, without evidence of previous acute fracture [5,8,9]. In 1888, German surgeon Franz Köning was the first to introduce the term OCD when discussing the etiology of intraarticular loose bodies [9,10]. It can be diagnosed in various joints, but the most affected are the knee, elbow, and ankle [11,12,13].

OCD of the elbow in OHAs primarily affects the humeral capitellum; however, the humeral trochlea, radial head, and olecranon have been rarely reported [14]. OCD of the elbow is usually diagnosed in male baseball players and female gymnasts, scarcely in more sedentary individuals [5,12,15]. The etiology of this disorder is believed to be multifactorial due to repetitive compressive forces, microtrauma, and localized subchondral ischemia following complex throwing biomechanics [15,16,17]. During physical examinations, patients often present with abnormal findings in the dominant arm, whereas the treatment options vary between conservative treatment and surgery, mainly depending on the patient’s age and symptoms and the location, size, and stability of the OCD lesion [8,18]. 

This narrative review aimed to comprehensively analyze the epidemiological data, etiological factors, clinical, imaging features, treatment options, and outcomes of OHAs with the diagnosis of elbow OCD. A literature search was conducted in PubMed/MEDLINE, Scopus, and Web of Science. There were no restrictions regarding the language or the year of publication. The search terms were “Osteochondritis dissecans”, “Elbow”, and “Overhead athletes”. Once a comprehensive list of abstracts was retrieved and reviewed, all studies were reviewed in full. Additional studies were identified by reviewing reference lists of selected articles.

## 2. Elbow Development and Biomechanics

The fully developed elbow arises from 6 secondary ossification centers [15]. According to a study by Tisano et al. [19], the capitellum emerges first between the age of 6–12 months. The epiphyseal plate of the radial head forms around the age of 3–4 years, whereas the medial epicondyle appears at the age of 5–7 years. The authors noted how these two centers tend to develop simultaneously in girls at the age of 5 years, while in boys, ossification centers tend to develop separately. Trochlea and olecranon originate at the age of 9–11 years, and the development concludes with the formation of the lateral epicondyle between 12 and 14 years of age. Fusion of the ossification centers starts with the capitellum, trochlea, and lateral epicondyle, followed by the olecranon, radial head, and medial epicondyle. As the final center fuses, the closing of the medial epicondylar apophysis concludes the skeletal maturity of the elbow between the age of 15 to 20 years. 

The standard overhead throwing cycle is divided into 6 phases: windup, early cocking, late cocking, acceleration, deceleration, and follow-through [20]. Tennis has additional phases in the cycle, such as racket preparation, acceleration, and follow-through for the forehand and backhand groundstrokes [16]. 

Heavy valgus and extension forces generated during the late cocking and early acceleration phases have a significant impact on the elbow [17,20]. The three main forces are associated with most elbow pathology: (a) medial tension forces with a focus on the medial epicondyle, (b) lateral compressive forces appearing along the radial part of the joint, and (c) posterior shear/compression forces along the posteromedial part of the elbow, mainly focused on olecranon [3,6,20]. At the elbow, valgus forces may reach up to 64 N-m [17]. Approximately 60% of compressive forces across the radiocapitellar joint are transmitted through the capitellum [21]. At the lateral radiocapitellar joint, compressive forces may reach up to 500 N [17]. In OHAs, besides compressive forces, the radiocapitellar joint is also transmitting repetitive shear forces, mostly during the late cocking and early acceleration stages [9].

## 3. Epidemiology and Etiology of Elbow OCD

### 3.1. Epidemiology

Overhead arm motions are severely utilized in many different sports. Therefore, OHAs are prone to elbow injuries, especially at a young age, which may impact their sport development later in life [6]. For instance, young pitchers account for 57% of all elbow injuries in high school baseball players [3]. Among tennis players, injuries sustained indoors have proven to be more severe compared to outdoor injuries, requiring a higher percentage of medical interventions [4].

Previous studies have shown that 32.9% of adolescent baseball players experienced elbow pain during throwing motions, whereas 81.7% of them reported episodes of prior elbow pain; however, they played through the pain, as they thought the pain was not severe enough to require medical assistance [9,10]. Another study found that 77.4% of young baseball players with confirmed capitellar OCD reported a positive history of elbow pain [10]. Furthermore, studies on young pitchers aged 9 to 12 showed that 25.5–28.0% of them reported elbow pain at least once during a season. However, 70% of these cases were categorized as mild pain that did not interfere with further competition [19]. Athletes who played through such elbow pain later presented with a higher grade of the osteochondral lesion [9]. Matsuura et al. [18] have found that age > 12 years, being in the pitcher or catcher position, and playing more than 100 games per year were significant risk factors for the development of elbow pain among young baseball players. There is an estimated 5% risk of severe injury for baseball pitchers between 9 and 14 years of age, defined as elbow or shoulder surgery, and/or retirement from the sport due to injury, within the 10-year interval [19]. When comparing pitchers and non-pitchers, one prospective study has shown that pitchers have a 4.5 times higher incidence of elbow injury [18]. Pitchers who were exposed to a high number of pitches per game (>80) during longer periods (>8 months per year) were four to five times more probable to sustain a shoulder or elbow injury that required surgical treatment [6]. Pitching in the setting of arm fatigue and high-velocity pitching (>85 mph) were also factors that increased the risk of shoulder or elbow injury [6].

There are several risk factors for the development of elbow OCD in OHAs, such as the male sex, sports involvement at a younger age, longer periods of competitive play, shorter throwers, and elbow pain at rest [1,3,5,9]. The estimated prevalence of capitellar OCD in baseball players varies between studies, and it is between 1.0 and 4.0% [1,7]. Trochlear OCD has been estimated to occur in 0.5–2.5% of OCD lesions of the elbow [22]. The incidence rate of OCD lesions also varies between studies and primarily depends on the age of patients. One study reported an incidence rate of 1.0–7.0% [5], whereas another study reported a 1-year cumulative incidence rate of 1.8% [23]. A group of authors from the United States of America conducted research on capitellar OCD lesions over a 25-year period [24]. After the implementation of inclusion and exclusion criteria, 45 individuals satisfied the requirements. Furthermore, 31 patients were divided into the younger group (10–15 years), whereas 14 patients were divided into the older group (16–24 years). They found the incidence rate of symptomatic capitellar OCD to be 6/100,000 overall, 9.5/100,000 for males and 2.6/100,000 for females. The detected incidence rates for the younger and the older group of patients were overall 10.6/100,000 and 3.1/100,000, respectively. 

Individuals with elbow OCD are usually 10–17 years of age [1,3]. According to recently published studies, the overall mean patient age ranged from 14.1 to 14.5. years [24,25,26]. The mean age of male and female patients was 15.1 and 12.5 years, respectively [24]. Due to a small number of documented trochlear OCD cases, the patient’s mean age varied greatly between studies: 13.4 [27] and 29.0 years of age [22]. Among individuals who were involved in overhead sports, OCD of the capitellum and/or trochlea was more frequently found in the male population [22,24]; however, some studies had more confirmed cases of OCD in the female population, including trochlear lesions [23,27]. Both capitellar and trochlear OCD were mainly detected in the dominant arm [22,23,24,27,28]. In patients with elbow OCD, sports involvement was reported in 66.7–90.5% of cases, and of these patients, 44.4–80.7% were OHAs [5,22,24], predominantly practicing baseball, gymnastics, football, and basketball [5,22,23,25,27,28,29].

To prevent serious injuries, there are recommendations for OHAs, primarily baseball players, and they include restrictions of no more than 8 months of overhead throwing per year, with at least 2–3 consecutive months of rest from throwing [3]. Furthermore, it is recommended not to pitch with fatigue or painful sensations, pitch consecutively on a daily basis, or pitch with the presence of any other body injury [3]. In Japan, guidelines were issued for baseball pitchers restricting individuals younger than 12 years to 50 pitches per day and 200 pitches per week [3]. According to the American Academy of Pediatrics recommendations, young athletes should be entitled to 2–3 months of scheduled rest a year, non-specific for the sport type [19]. In general, the education of athletes, their families, and coaches, followed by restrictions in the count of overhead motions, can be helpful in the prevention of any type of injury or reinjury of the elbow [15].

### 3.2. Etiology

Chronic elbow injuries are essentially caused by overuse and repetitive stress placed on the developing elbow joint of the dominant arm, most frequently in OHAs [6,15,30]. Taking into consideration these circumstances, a typical elbow lesion is capitellar, rarely trochlear OCD [9,21]. It is presumed that OCD lesions have multifactorial causes, such as repetitive trauma applied on the radiocapitellar joint, the biomechanical disproportion of the radial head and capitellum, poor capitellar vascular supply, and inflammatory and genetic factors [9]. OCD lesions of the capitellum are usually located centrally or laterally. In addition, in baseball pitchers, lesions are frequently found in the anterior part of the capitellum [7,21]. OCD lesions of the trochlea are typically found in the posterior parts of the lateral trochlea [22,31].

Biomechanical studies have shown certain disproportions in capitellar hardness, which decreases from medial to lateral, accompanied by a soft medial part of the radial head and increased stiffness of the central part of the radial head [9,21]. Subsequently, the central part of the radial head is significantly stiffer compared to the lateral part of the capitellum, which is a substantial risk factor in the setting of increased loading activities [9]. No significant difference was found in the stiffness of the radial head and the medial part of the capitellum [21]. Furthermore, observations of the radial head enlargement are still debatable whether it is an etiological factor or a sequela of capitellar OCD [21], whereas radial head lag during elbow motion with subsequent radiocapitellar incongruency has been proposed as a novel biomechanical cause of OCD [32]. Trochlear OCD lesions in throwing athletes are assumed to be the result of olecranon abutment due to micro instability caused by collateral ligament insufficiency or laxity [31].

The poor vascular supply of the humeral capitellum is proposed to be another etiological factor for OCD lesions. Capitellar arterial blood supply depends on the small posterior perforating branches arising from radial recurrent, radial collateral, and interosseus recurrent arteries [9,16,33]. However, these vessels lack sufficient collateral branches with the metaphyseal vessels, forming a watershed bone region with a tenuous blood supply [9,33]. As time progresses, repetitive trauma leads to the disruption of blood flow and local ischemia, followed by the development of osteochondral alterations in the subchondral bone and the formation of the intraarticular loose bodies [9,16,33]. The inferior portion of the lateral trochlea is also a relative watershed region due to the scarce vascular supply between the non-overlapping medial and posterior vascular arcades [31].

Lateral compressive forces across the capitellum may lead to osteochondral damage and loose fragments, while shear forces may lead to alterations of the posteromedial tip of the olecranon and trochlea/olecranon fossa, resulting in the formation of osteophytes, loose bodies, and fractures [17]. These types of changes can be objectified on pathohistological images and classified into OCD-type IA, almost preserved cartilage; OCD-type IB, cartilaginous degeneration; OCD-type IIA, cartilage ossifying; and OCD-type IIB cartilage osteonecrosis [25]. 

## 4. Clinical Presentation

Athletes with predominantly capitellar OCD typically presented with the onset of laterally localized, dull, progressively worsening, and activity-related pain of the elbow [1,6,9,15,16,34]. Elbow pain was also the most common symptom in patients with trochlear OCD (93–100% of cases) [22,27,35]. Mechanical symptoms, such as catching, locking, or clicking in specific positions, were also characteristic of elbow OCD and raised suspicion for intraarticular loose bodies or articular instability [3,6,9]. In OHAs, the dominant arm was almost always affected [9,10]. One study reported that the median duration of symptoms, prior to the surgical intervention of capitellar OCD, was 16.2 months (range 2 weeks to 9 years) [5]. The presenting symptoms of trochlear OCD lasted between 7 days and almost 3 years (mean 5.6 months) [27] and, in another study, between 2 weeks to 1 year (mean 6.8 months) [22].

During the physical examination, swelling, stiffness, palpatory tenderness, and crepitations of the elbow were also encountered [1,3,5]. Crepitus was often associated with elbow supination and pronation [9,10]. A positive radiocapitellar compression test was indicative of OCD due to pain at the radiocapitellar joint after active pronation and supination in an extended elbow [9]. However, patients with OCD lesions also experienced pain during the palpation of the radiocapitellar joint or capitellum when the elbow was fully flexed [3,10]. On elbow evaluation, a limited range of motion (ROM) in pronation, supination, and extension was noticed, most dominantly a 15–30° decrease in extension [1,3,5,9,10,34]. According to a study by Wang et al. [27], 32% of patients with trochlear OCD, who were in majority OHAs, presented with a reduction in elbow mobility, such as flexion contractures (mean 11°, range 5–20°) and extension contractures (mean 20°, range 5–35°). 

## 5. Imaging

Imaging methods for the assessment of elbow OCD include radiography, computed tomography (CT), magnetic resonance imaging (MRI), and ultrasound (US). Capitellar OCD lesions can be localized as central (within the articular surface of the capitellum, 51.8%) or lateral (extension to the lateral cortex of the capitellum, 48.2%) [36]. Furthermore, in OHAs, lesions are frequently found anterolaterally [9,37], sometimes in the posterolateral zone [38]. There are reports that lateral lesions were significantly larger than central lesions [39]. According to a study by Wang et al. [27], trochlear OCD lesions can be divided into typical (inferior trochlear articular surface) or atypical (posteromedial trochlear articular surface). 

**Figure 1:**
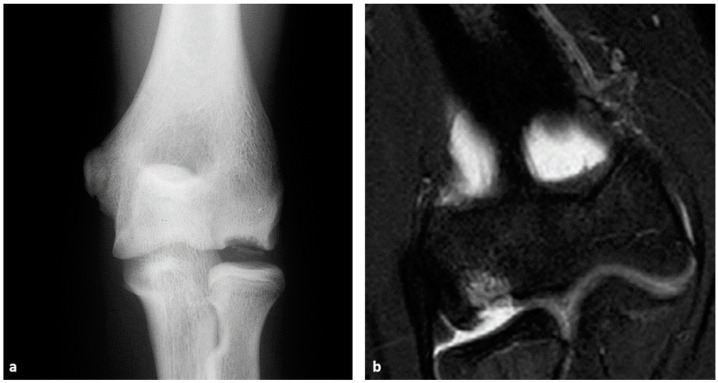
OCD lesions in different patients: (**a**) Anteroposterior X-ray view of the elbow—an irregular capitellum with a radiolucent defect with sclerotic contours and fragmentation of the articular surface; (**b**) PD-weighted fat-suppressed coronal MR image—full-thickness chondral defect of the capitellum with edema of the subchondral bone.

Radiography is usually the initial method for the evaluation of OCD lesions in the elbow (Figure 1a). However, early-stage OCD is easily missed in these images [40]. Anteroposterior, lateral, and oblique projections are typical projections for the radiographic assessment of the elbow, whereas the addition of an anteroposterior view with a 45° flexion of the elbow may improve the visualization of the lesion [1]. Characteristic findings of capitellar OCD lesions are subchondral irregularity or lucency of the capitellum, with or without the signs of sclerosis, cortical fragmentation, and/or intraarticular loose fragments [1,9,16,17]. Similar radiographic features are displayed in OCD lesions of the trochlea [22,31]. The widely used radiography classification for the assessment of OCD lesions is the Minami classification [10], and others include Bruns, Berndt and Harty, Matsuura, Kida, Takahara, Anderson, and many other classifications [21,40,41]. Claessen et al. [40] have found the Minami classification to be the most reliable, however, with only fair inter-observer agreement among orthopedic surgeons and musculoskeletal radiologists. Takahara et al. [25] have confirmed that radiographic OCD stages I (radiolucency or slight calcification) and II (delayed ossification or bony fragment) have a substantial correlation with pathological OCD-I (cartilaginous), and OCD-II (osteochondral) lesions. The risk for developing arthritis of the radiocapitellar joint is present in patients with larger, laterally located capitellar OCD lesions found on radiographic images, especially with an addition of pathomorphological changes of the medial epicondyle of the humerus [42]. Regarding the intra- and interobserver agreement, one study demonstrated only fair-to-moderate agreement between seven orthopedic surgeons detecting elbow OCD lesions on radiographs [43]. The reported overall sensitivity of radiography in the detection of OCD lesions is between 44–47% [33].

CT features of elbow OCD lesions are equivalent to those on radiography, yet CT provides a more detailed depiction of osteochondral abnormalities. A subchondral hypodensity of the capitellum with variations in the degree of bone sclerosis and fragment attachment are characteristics of OCD lesions [17]. Compared to radiography, the major advantage of CT is the detection of subtle osteochondral injuries with enhanced visualization of the detached fragment and intraarticular localization [10,17]. Therefore, CT is also recommended in preoperative planning. The greatest fragment diameter of more than 8 mm and/or a sclerotic rim thicker than 3 mm are highly suggestive of fragment instability [17]. In the study by Collins et al. [22], almost all patients (eight/nine) with trochlear OCD have demonstrated the presence of small osseous fragments, with six of them being in situ fragments. The often-used classifications for the CT staging of capitellar OCD are Ferkel and Sgaglione and Clanton and Lee [41]. A group of international authors introduced quantitative 3D-CT with heat-mapping techniques for the evaluation of capitellar OCD in OHAs primarily involved in baseball, gymnastics, and basketball [38]. The median OCD surface was 101 mm² with great intra- and interobserver agreement. Using heat-mapping, the authors demonstrated that the posterolateral zone of the capitellum was most frequently damaged, and individuals with OCD lesions involving lateral parts generated larger lesions, had longer-lasting symptoms, and showed limited ROM during elbow extension. A group of Japanese authors also used advanced imaging methods for the preoperative assessment of elbow OCD [44]. They used 3D MRI-CT fusion images for lesion evaluation and accurately matched the International Cartilage Repair Society (ICRS) classification in 93.5% of patients.

Compared to other imaging methods, since 1995, MRI has slowly emerged as a mainstay for the evaluation of elbow OCD [24]. It is especially useful in the early stages of the disease in comparison with other imaging techniques. According to Anderson et al. [20], the standard imaging protocol includes scanning in the supine position using a surface coil with an elbow located at a side. Images are acquired in axial, coronal, and sagittal planes with the use of T2-weighted (T2W), T1-weighted (T1W), and/or proton density (PD) sequences; T2W and PD sequences are usually combined with the fat-saturation (FS) technique (Figure 1b). The earliest MRI features of OCD lesions in the elbow are uniform hypointensity of the superficial capitellum on T1W images with normal capitellar morphology on T2W images [9]. As the lesion progresses, changes are detectable on both T1W and T2W sequences [9,33]. Subchondral edema of the capitellum on FS T2W images is often a distinguishable secondary feature [20]. Complete or partial (crater-like) contrast enhancement of the OCD lesion following gadolinium administration is a favorable finding that suggests preserved vascularity and the viability of the fragment [9,17]. However, marginal or no contrast enhancement of the fragment is associated with instability and unsatisfactory clinical outcome [17]. Features highly suggestive of capitellar fragment instability are defects of the articular surface; thin, circumferential hyperintense T2W signal between the OCD lesion and subchondral bone; hyperintense, linearly formed T2W signal through cartilage; high signal cystic formations underlying the OCD lesion on T2W images; and/or a clearly visible displacement of the fragment [9,16,17,31]. In the study by Nguyen et al. [45], the authors evaluated elbow OCD lesions in children and found that an osteochondral defect, intraarticular loose body overlying cartilage changes, subchondral plate disruption, and hyperintense rim signal were more commonly observed in unstable than stable OCD lesions. However, only osteochondral defects and an intraarticular loose body were typical of OCD instability, with 100% specificity. MRI features of trochlear OCD lesions are similar to those of the capitellum [31]; however, Wang et al. [27] have found additional accompanying abnormalities to trochlear lesions. The most common findings were capitellar OCD or capitellar edema, and one patient had capitellar and coronoid OCD lesions. Several classifications are commonly used for the preoperative evaluation of capitellar OCD: Nelson, Itsubo, Hefti, Dipaola De Smet, and Kohyama classification [37,41]. In the study by Iwasaki et al. [37], De Smet and Dipaola classifications a demonstrated sensitivity and specificity of 89/44% and 83/44%, respectively, for the assessment of fragment instability. The authors also reported that the overall correlation rate between the Dipaola classification and intraoperative staging was 41%, leading to their conclusion that MRI is still ineffective in predicting the intraoperative stability of OCD lesions. However, Kohyama et al. [8] developed a four-stage MRI classification for elbow OCD, mainly focusing on the outline of the capitellum and articular cartilage status. It showed an agreement in 88.9% of cases with the International Cartilage Repair Society (ICRS) classification used for the intraoperative staging of OCD lesions. Furthermore, their classification displayed a sensitivity, specificity, positive predictive value (PPV), and negative predictive value (NPV) of 98.4, 84.2, 95.3, and 94.1%, respectively, for the identification of unstable OCD lesions. Based on a systematic review and meta-analysis by Hu et al. [11], the sensitivity, specificity, and area under the curve (AUC) value of MRI in assessing the stability of OCD lesions (23% of them in the elbow) was 92%, 85%, and 0.95 respectively. In the evaluation of the stability of juvenile OCD lesions, the sensitivity, specificity, and AUC values of MRI were 93%, 68%, and 0.92, respectively. 

There has been a proposal for the US to be primarily used as a screening method, especially for the detection of early-stage OCD lesions in the elbow due to reported PPV values ranging from 67 to 100% [1,9]. Although Yoshizuka et al. [46] reported good results using the US in the evaluation of capitellar OCD, further studies are still needed to confirm these findings. One of the used US classifications for the diagnosis of elbow OCD is Ishizaki and Yang classification [41]. Furthermore, earlier reports also showed that the US is a highly accurate imaging modality for the detection of intraarticular loose bodies in the elbow (100% sensitivity, 95% specificity) [17].

In a systematic review by Pu et al. [41], the authors suggested the following imaging guidelines for the evaluation of capitellar OCD lesions. First, a patient with elbow pain or suspicious findings on US screening should obtain the elbow X-ray scans assessed according to the Minami classification. The OCD lesion instability should be evaluated on MRI images using the Kohyama classification. CT should be used for the detection or confirmation of subchondral fragmentation and intraarticular loose bodies as well as preoperative planning. 

## 6. Differential Diagnosis

In the OHA population, it may be difficult to distinguish elbow OCD lesions from other pathologic conditions due to the similarity in clinical and imaging features. The main pitfalls in OCD diagnosis are Panner disease, the pseudo-defect of the capitellum, and inflamed posterolateral plica of the elbow [17,33]. 

Panner disease is a self-limiting osteochondrosis of the capitellum and the most common cause of lateral elbow pain in the population younger than 10 years of age [19,33]. The etiology of this condition is yet to be discovered; however, one of the causes is believed to be chronic ischemia to the small posterior perforating end arteries of the capitellar epiphyseal cartilage [31]. Patients often present with laterally localized, dull elbow pain, stiffness, and swelling, accompanied by the progressive worsening of the pain following activity [31,33]. On plain radiographs, the capitellum is entirely affected by Panner disease, appearing to decrease in size, appearing fragmented, and displaying zones of sclerosis and irregularity in capitellar margins [9,31,33]. The capitellar ossification center demonstrates a hypointense signal on T1W images and a variable fluid-sensitive MRI signal depending on the stage of the disease [31]. Bone marrow edema may be present in both OCD lesions and Panner disease [19]. Panner disease is not associated with chondral pathology; therefore, the prognosis is more than favorable, often demanding only conservative treatment, without any long-term sequelae [31,33]. In most patients, the resolution of symptoms usually occurs after 6–8 weeks of rest [9]. In opposition to Panner disease, there are several differentiating elements favoring OCD of the elbow: patients between 12 and 17 years of age, noticeable damage of the cartilage, intraarticular loose bodies, mechanical symptoms, and anteriorly located focal capitellar lesion [16,19].

The pseudo-defect of the capitellum can be visualized on MRI as a false indentation on the posterolateral margin of the capitellum near the junction with the adjacent anterolateral capitellum [17,31]. It is often mistaken for an OCD lesion [17]. Knowing the location of this structure in the elbow and the use of sagittal planes may be useful to avoid the overdiagnosis of OCD lesions, which are typically located more anteriorly, following the curved articular surface of the capitellum [17,31]. An inflamed posterolateral plica is also associated with lateral elbow pain, swelling, and palpatory tenderness, however, with unremarkable radiographs or MRI visible capitellar changes [33].

## 7. Treatment and Outcome

### 7.1. Trochlear OCD

Trochlear lesions of elbow OCD may be caused due to a different overload pattern than capitellar OCD [22,27]. Apart from this, trochlear OCD is in 68% of cases found with a coexisting elbow pathology and is rarely found as an isolated lesion [27]. This, in addition to the localization of the lesion on the lateral trochlear ridge, is the most important indication for surgery in the general population. Collins et al. reported a series of 10 patients, 6 of whom were athletes, with trochlear OCD on the lateral trochlear ridge, with a mean age of 29 (range, 15 to 58) years. In 9 out of 10 cases, arthroscopic surgery was performed, while conservative management was preferred in a single patient [22]. Of these nine patients that required arthroscopy, four were treated with osteophyte and/or loose body removal, three with debridement and microfracture, and one with chondroplasty and loose body removal. At the mean follow-up of 7 (range, 1 to 30) months, the resolution of elbow pain and improvement of mechanical symptoms were reported in seven out of eight available patients.

On the other hand, Wang et al. reported more frequent conservative treatment in 28 adolescent athletes with trochlear OCD of the elbow that were 13.4 ± 1.6 years old [27]. The initial treatment consisted of physical therapy and avoiding overhead sporting activities for 3 months. In patients with an acute onset of symptoms, within 2 weeks, an unlocked hinged brace was used at the beginning as well. The progression of radiological signs of OCD was present in four elbows (14.3%) on an MRI that was performed at an average of 12 (range, 5–31) months after starting the treatment. These were addressed with surgery, with drilling, or with curettage in three patients and the fixation of the fragment in one patient. At the average follow-up of 12 (range, 9–15) months after surgery, three of the four patients had improvement regarding pain.

### 7.2. Capitellar OCD

The correct approach in the treatment of capitellar OCD of the elbow is based on the guidelines proposed by Takahara et al. [47] to determine whether the osteochondral fragment is stable or not. These guidelines consider the closing of the physeal plate of the humeral capitellum, the ROM of the elbow, and the radiological markings of fragment stability [47] (Table 1). According to Takahara, conservative treatment should be performed in cases of stable OCD, which is very rare. Surgical treatment, on the other hand, should be performed in any cases of instability of OCD, cases with a present loose body, or after 6 months of conservative treatment that is not successful [47,48].

#### 7.2.1. Conservative Treatment

The cornerstone of conservative treatment is avoiding activities that cause pain in the elbow during a period of 3 to 6 months. Some authors also suggest a short period of elbow immobilization in a hinged elbow brace at the beginning of the treatment [9]. Results from the literature suggest that this kind of treatment is successful only in the early stages of the disease when the osteochondral fragment is stable [21,49,50,51]. Mihara et al. [52] published a study that showed healing of the fragment at an average 14.4 (range, 6 to 56) months follow-up in 94% (16/17) of patients who initially had stable OCD and were treated conservatively for 6 months. On the other hand, conservative treatment resulted in the healing of the fragment in only 50% (11/22) of patients with initially unstable OCD [51].

#### 7.2.2. Surgical Treatment

Surgical treatment was previously based on the removal of the damaged cartilage and the underlying subchondral bone and was performed with open surgery [47,53,54,55,56,57]. The results of this type of surgery suggested that the patients may still have residual pain, a limited ROM, and degenerative changes of the elbow after surgery [58,59]. To enhance the results of surgical treatment, Kuwahata and Inoue published a study in 1998, describing the treatment of OCD of the humeral capitellum with open grafting of the defect with autologous cancellous bone and screw fixation of the osteochondral fragment [60]. On the other hand, with the development of arthroscopic surgery, Baumgarten et al. described the arthroscopic removal of the osteochondral fragment with abrasive chondroplasty of the underlying subchondral bone, while Bojanic et al. described the removal of the fragment and microfracture technique [54,61,62,63]. Furthermore, a trend toward the treatment of cartilage defects with osteochondral autograft transfer (OAT) became more and more popular in different joints, including the elbow [12,64]. 

Nowadays, the surgical treatment of capitellar OCD of the elbow can be divided into three groups of techniques. These include either bone marrow stimulation (BMS) techniques, fragment fixation techniques, or regenerative surgical treatment. The last group includes the OAT technique, new generations of autologous chondrocyte implantation (ACI), osteochondral allograft transplantation, and transplantation of the tissue-engineered cartilage [7,38,47,53,54,55,56,57]. While most of the BMS and fragment fixation techniques are nowadays performed with arthroscopic surgery, most of the regenerative techniques still demand open surgery. In general, the surgical treatment of capitellar OCD results in high rates of return to sports in children and adolescents. According to the meta-analysis by Cohen et al. of 31 studies that included both open and arthroscopic surgeries, a return-to-sport for overhead athletes was 89.4%, while the rate of return to any sport was 97.6% [5].

Definitive guidelines for the surgical treatment of OCD of the humeral capitellum are still not determined due to a lack of long-term follow-up studies comparing different techniques. Furthermore, the lack of a universal classification system, the inability to determine a return-to-sport level, and the high number of different scores used for clinical assessment of the elbow also make the comparison between different techniques difficult. Nevertheless, Kolmodin and Saluan suggested that the type of treatment should be determined based on the localization of the capitellar OCD, i.e., if the lateral edge of the articular surface of the humeral capitellum is part of the lesion [38,65]. It has been suggested that patients with capitellar OCD reaching the lateral edge have worse results, more frequent extension deficit, and swelling of the elbow [66,67]. The main reason for this is the shear forces that are present on the lateral edge, making the healing more difficult, as opposed to the compressive forces that are present in the central part of the capitellum, thus enhancing the healing [67]. Therefore, it is suggested that a BMS or fragment fixation techniques may be used with central capitellar OCD, while in cases where the OCD includes the lateral edge, the OAT technique may be a better choice [26,68]. Furthermore, according to Funakoshi et al., predictors of unsuccessful surgical treatment may be radial head enlargement and the advanced skeletal age of the throwing side compared with that of the nonthrowing side on the preoperative radiographs [69].

#### 7.2.3. Bone Marrow Stimulation

**Figure 2:**
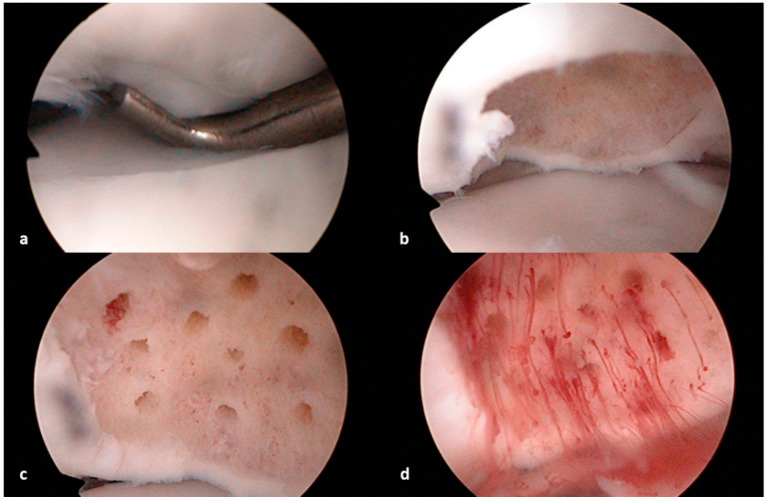
Arthroscopic treatment of osteochondral defect of humeral capitellum using the microfracture technique; (**a**)—arthroscopic visualization of the osteochondral defect using the arthroscopic probe to determine the edges of the defect, (**b**)—intraoperative image after the removal of the osteochondral fragment and debridement of the sclerotic subchondral bone, (**c**)—intraoperative image after performing the microfracturing of the subchondral bone with an arthroscopic awl, (**d**)—intraoperative image after the inflow of the sterile saline to the elbow is stopped and the suction turned on, showing good bleeding from the healthy subchondral bone.

**Figure 3:**
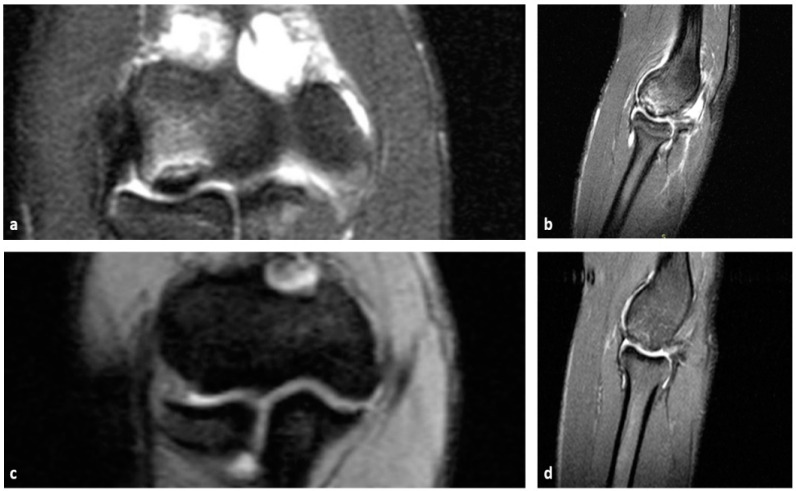
Preoperative coronal and sagittal PD fat-saturated MR images showing an unstable osteochondral defect (**a**,**b**). Postoperative coronal and sagittal PD fat-saturated MR images showing a satisfactory surgical outcome (**c**,**d**).

Bone marrow stimulation (BMS) can be achieved with either abrasive chondroplasty, drilling, or the microfracture technique [54,61,62]. The main goal of these techniques is to make an opening to the healthy subchondral bone after the removal of the osteochondral fragment. This way, blood from the subchondral bone is expected to stimulate the filling of the defect with fibrocartilaginous tissue, similar to the hyaline cartilage. Abrasive chondroplasty uses a motorized instrument to remove the calcified, sclerotic layer of the subchondral bone under the defect to reach the healthy, well-vascularized subchondral bone. On the other hand, with drilling and the microfracture technique, small holes are made through the subchondral bone perpendicular to its surface (Figure 2). The downside of the drilling technique is thermal damage to the edges of the subchondral bone, while this risk does not exist with the microfracture technique. With this technique, a special instrument called a microfracture awl is used to make the holes via the manual compression of the instrument into the subchondral bone [54,62]. Regarding the available literature, the removal of the osteochondral fragment and arthroscopic microfracture of the subchondral bone is the technique that is most used, with good results (Figure 3, Table 2). According to a study by Obey et al. [70], MRI can be used to determine the healing of the cartilage tissue repair following arthroscopic debridement and BMS. Patients who subsequently demonstrated capitellar subchondral edema were more likely to be reoperated.

In addition to the BMS technique, augmentation by the implantation of ultra-purified alginate (APUL) gel has also been described [86]. The authors performed open surgery of the elbow in five athletes with capitellar OCD, performing the BMS with drilling and filling the defect with APUL. Afterward, CaCl_2_ was injected on the surface for gelation and was washed out 5 min later with normal saline. At a mean follow-up of 97.2 (range, 96–99) weeks, all patients returned to competitive-level sports, while four patients were pain-free with excellent improvement regarding the clinical score. Although these short-term results are encouraging, further studies are needed to confirm such procedures on a higher number of patients.

#### 7.2.4. Fixation of the Osteochondral Fragment

The goal of this treatment method is to stabilize the unstable osteochondral fragment in the subchondral bed and thus enhance bone healing. Different fixation techniques and devices have been used until today [55,57,58,59,60,87,88,89] (Table 3). In all cases, it is advised to use fixation if the fragment is not fragmented or dislocated from the subchondral bed [55,57,58,59,60,87,88,89].

#### 7.2.5. OAT

Regenerative techniques were developed with the purpose of achieving the presence of hyaline cartilage at the area of the OCD, instead of achieving fibrocartilaginous tissue as with BMS techniques. The first such treatment of elbow OCD was described by Tsuda et al. in 2005 [68,92]. The most-used technique is the OAT technique, which enables the transplantation of not only the cartilage but also the subchondral bone, which is important for transplant healing and mechanical resistance to load. The most common donor sites are the knee and the rib, i.e., the 5th and 6th rib [12,29]. The transplant from the knee is taken from the edges of the femoral trochlea, often as part of the mosaicplasty technique [12,39]. Using this donor site has some limitations when a larger defect of the humeral capitellum is present because it is not advised to take transplants larger than 9 mm in diameter each [93]. In such cases, multiple transplants need to be taken from the knee to perform mosaicplasty. A good available alternative is to take a single osteochondral transplant from the rib, which can be up to 20 mm in diameter. 

The downside of the OAT technique is the need for open surgery of the elbow as well as possible donor site morbidity. Regarding the knee, it can be found in 7.8% of cases and is mostly due to pain, swelling, or a clicking sensation [68]. On the other hand, donor-site morbidity is not as common in the ribs and can be found in 1.6% of patients, with donor-site pain being the common complication [68]. Nevertheless, Shimada et al. described a serious life-threatening complication of pneumothorax due to a lesion of the parietal pleura in a single patient after taking an osteochondral transplant from the rib [94]. Despite these possible complications, the OAT technique provides excellent functional postoperative results according to the literature [64], which is comprehended in Table 4.

#### 7.2.6. ACI

Autologous chondrocyte implantation (ACI) was a surgical technique first developed to treat OCD lesions of the knee and ankle. It implies arthroscopic harvesting of the healthy cartilage in one stage of surgery, the in vitro isolation and cultivation of the chondrocytes, and the implantation of these chondrocytes in the second stage of surgery. The main advantages of such a technique are the preservation of the hyaline cartilage at the site of the OCD and the ability to perform both surgeries arthroscopically. On the other hand, the drawbacks are the need for two surgeries and no restoration of the subchondral bone, as opposed to the OAT technique. Regarding the elbow, only a few case reports have been described in the literature, with Sato et al. being the first one in 2004 [48,110,111,112]. While Sato et al. showed excellent results at a 2-year follow-up in a single patient, Iwasaki et al. described two cases of capitellar OCD treated with ACI, with the improvement of the ROM of the elbow and Mayo Elbow Performance Index scores at the 4-year follow-up [48,110].

#### 7.2.7. Allograft

The main idea of fresh osteochondral allografts (OCA) in the treatment of capitellar OCD is to avoid the donor-site morbidity that is possible in the OAT technique as well as to avoid surgery on the healthy extremity. The graft can be taken from either a cadaveric capitellum or femoral condyle [10]. Until today, only a few papers have been published regarding this matter [113,114,115]. Mirzayan and Lim published a report of 9 cases of baseball players with capitellar OCD treated with OCA [114]. The mean follow-up was 48.3 (range, 11–90) months, after which significant improvement in functional scores was achieved. All patients returned to throwing activities and were either still active in sports or played baseball for at least 2 years after surgery before leaving the sport regardless of the operated elbow [114]. A recent study by a group of authors from the United States of America tried to identify the best femoral graft site for repairing capitellar OCD lesions based on the measurement of the radius of curvature of the most common localization for capitellar OCD and different areas of potential donor femoral condyle grafts. After 15,000 CT scan simulations of condylar-to-capitellar site matchings, they concluded that an adequate graft was achieved in 15% of cases, with a graft from the epiphyseal scar area being the closest match [115].

## 8. Rehabilitation

The rehabilitation of the OHAs after elbow OCD can be divided into 4 phases: (a) healing/immobilization, (b) the recovery of ROM, (c) strengthening, and (d) sport-specific activity [10]. In the first phase, it is particularly important to reduce pain and inflammation and slow down muscle atrophy [19]. If needed, pain and inflammation may be reduced by using cryotherapy, laser, and high-voltage stimulation [116]. A systematic review and meta-analysis by Cohen et al. [5] showed that in the majority of studies, postoperative immobilization lasted for 1–4 weeks, usually 2 weeks, with the arm placed in a neutral position in the above-elbow splint. In the review by Logli et al. [10], the authors reported that elbow immobilization is not regularly used following arthroscopic debridement or the microfracture treatment of capitellar OCD lesions. After osteochondral autograft transfer for capitellar OCD lesions, Patel et al. [64] suggested elbow immobilization until the first follow-up examination. 

At the onset of the second phase, moist heat, warm whirlpool, and ultrasound can be used to prepare soft tissues for stretching and to enhance the extensibility of the elbow capsule, muscles, and tendons [116]. In the second phase of rehabilitation, the primary goal is to restore elbow ROM by 6–8 weeks postoperatively [10]. In their comprehensive review, Wilk et al. [116] emphasized the importance of early ROM activities in OHAs due to positive effects on articular cartilage and the synthesis, alignment, and organization of collagen fibers. Furthermore, the authors noted that ROM exercises are used to prevent scar tissue formation and adhesions, thus preventing the contracture of the elbow [116]. When BMS techniques are used for the treatment, rehabilitation usually starts on the second postoperative day [54]. In the first several weeks, the focus is on regaining ROM using a continuous passive motion technique, later followed by active motion assisted by physiotherapists [54]. 

A strengthening program can be initiated when the full ROM, minimal pain, and tenderness and a good (≥4/5) manual strength test of the elbow flexors and extensors are accomplished [10,116]. Strengthening exercises include isotonic contractions, at the beginning concentric, later eccentric, with an emphasis on elbow flexion and extension, wrist flexion and extension, and forearm pronation and supination [116]. In patients following the OAT technique for capitellar OCD lesions, a light strengthening program is initiated at 6 weeks postoperatively [64]. 

The sport-specific activities, therefore, the advanced strengthening phase, may begin after the list of criteria is fulfilled: full non-painful internal and external rotation, total ROM, non-present pain or tenderness, and strength replicating 70% of the contralateral extremity strength [116]. During this phase, exercises include gradual progression to higher resistance, functional movements, plyometric activities, and eccentric contraction, while plyometric exercises are particularly beneficial for elbow rehabilitation in OHAs [116]. Following microfracture and simple debridement procedures, patients usually return to sport-specific activities around 3 months postoperatively [10]. In more regenerative procedures due to larger OCD lesions of the elbow, return is delayed until the visualization of bone healing on imaging methods, usually around 6 months postoperatively [10,34]. Therefore, the full return to competition is expected no earlier than 6 months postoperatively, with no throwing or push-ups advised until 3 months postoperatively [5,10,64].

## 9. Conclusions

OCD lesions are a significant problem in sports, especially in OHAs, and are usually found at the humeral capitellum. The mainstay for the diagnosis of elbow OCD is MRI. Treatment of elbow OCD lesions may be conservative or surgical. Conservative treatment is successful only in the early stages of the disease when the osteochondral fragment is stable. Surgical treatment is reserved for unstable OCD lesions or unsuccessful conservative treatment. There is a variety of surgical procedures mainly depending on the localization and the size of the OCD lesion. Adequate procedure selection ensures excellent results and a prompt return to sports activities. 

The most important methods in the prevention of serious injuries in OHAs are the detection and monitoring of risk factors, following sport-specific workload guidelines, executing appropriate elbow biomechanics, and planning age-specific strength and condition programs. Therefore, medical practitioners should be especially aware of OHAs presenting with elbow pain and possible OCD lesions to provide adequate evaluation and favorable outcomes. 

## Figures and Tables

**Table 1:** Modified classification of capitellar OCD of the elbow adapted from Takahara et al. [47].

Osteochondral Fragment	Humeral Capitellum Growth Plate	Range of Motion in the Elbow	Radiological Markings	Treatment
Stable	Open	Full	Flattening or radiolucency of the subchondral bone	Conservative
Unstable	Closed	Extension deficit ˃ 20°	Fragmentation of the osteochondral lesion	Surgical

**Table 2:** Literature review of studies including general information about patients, surgical technique, and results after arthroscopic treatment without fragment fixation of capitellar osteochondritis dissecans of the elbow.

First Author/Year of Publication	Number of Patients (Number of Elbows)	Male/ Female (N)	Mean Age (Range) at the Time of Surgery	Type of Arthroscopic Treatment	Follow-Up (Range) (Months)	Mean Score Value before Surgery/Mean Score at Final Follow-Up (Used Score)	Return to Sports at the Same or Higher Level as before Surgery (%)
Baumgarten/1998 [61]	16 (17)	12/4	13.8 (10–17)	Abrasion chondroplasty	48 (24–75)	N/A	81
Ruch/ 1998 [71]	12 (12)	10/2	14.5 (8–17)	Debridement	39 (24–70)	N/A	92
Byrd/ 2002 [72]	10 (10)	10/0	13.8 (11–16)	Debridement or abrasion chondroplasty	47 (36–72)	N/A/194 (T&A ^1^)	40
Brownlow/2006 [73]	29 (29)	20/9	22 (11–49)	Debridement	77 (7–149)	N/A	78
Bojanić/2006 [62]	3 (3)	1/2	14 (13–15)	Microfracturing	16 (14–18)	N/A/97 (MEPS ^2^)	100
Rahusen/2006 [74]	15 (15)	6/9	28 (16–49)	Debridement	45 (18–59)	65.5/91 (MAESS ^3^)	80
Schoch/ 2010 [75]	13 (13)	10/3	16 (10–25)	Drilling	43 (12–96)	N/A/8.6 (DASH ^4^)	NA
Miyake/2011 [76]	106 (106)	105/1	15 (12–18)	Drilling	13 (8–46)	N/A	85
Bojanić/ 2012 [54]	9 (9)	6/3	15 (12–19)	Microfracturing	60 (24–108)	53/98 (MEPS ^2^)	89
Tis/ 2012 [77]	12 (13)	7/5	13.1 (10–14)	Drilling	23 (2–60)	N/A/196 (T&A ^1^)	58
Wulf/ 2012 [78]	10 (10)	6/4	13.9 (10–18)	Microfracturing	42 (27–54)	70.5/97 (MEPI ^2^) 116/193 (T&A ^1^)	75
Lewine/2016 [79]	21 (21)	13/8	13.4 (NA)	Drilling (66.7%) or microfracturing (33.3%)	26 (NA)	156/184 (T&A ^1^)	67
Bexkens/2017 [80]	71 (75)	30/41	16 (11–26)	Microfracturing	42 (12–98)	N/A/40.8 (OES ^5^)	55
Ueda/2017 [81]	38 (38)	33/5	14 (13–15)	Debridement	96 (60–144)	62.7/92.3 (JOA ^8^)	100
Matsuura/2020 [82]	23 (23)	NA	14.7 (13–17)	Debridement (56.2) or drilling (43.8%)	138 (120–156)	160/195 (T&A ^1^)	87
Ueda/2021 [83]	19 (19)	17/2	14 (13–15)	Debridement	96 (60–132)	129/182 (T&A ^1^) 46/3 (DASH ^4^)	95
Michelin/2022 [26]	17 (18)	14/3	14.1 (11–17)	Drilling or microfracturing	52.8 (24–120)	N/A/94.1 (KJOCS ^6^) NA/2.3 (QuickDASH ^4^)	66.7
Austin/2023 [84]	49 (53)	38/11	15 (11–18)	None (21%) or debridement or drilling or microfracturing ^a^	132 (60–276)	N/A/4 (QuickDASH ^4^)	80
Rothermich/2023 [85]	90	66/24	15.2 (11.4–43.1)	Debridement or abrasion chondroplasty or drilling or microfracturing or stem cell implantation	99.6 (NA)	N/A/87.1. (AC ^7^) NA/83.5 (KJOCS ^6^)	93

^1^ Timmerman and Andrews elbow scores; ^2^ Mayo Elbow Performance Score; ^3^ Modified Andrews Elbow Scoring System; ^4^ Disabilities of Arm, Shoulder, and Hand; ^5^ Oxford Elbow Score; ^6^ Kerlan-Jobe Orthopaedic Clinic Score; ^7^ Andrew/Carson score; ^8^ Japanese Orthopaedic Association score; ^a^ In 21% of patients, the capitellum humeri appeared healed and no further surgical intervention was performed. N/A = unknown.

**Table 3:** Literature review of studies including general information about patients, surgical technique, and results after arthroscopic fixation of the osteochondral fragment in patients with capitellar osteochondritis dissecans of the elbow.

First Author/Year of Publication	Number of Patients (Male/Female)	Mean Age (Range) at the Time of Surgery	Type of Fixation of the Osteochondral Fragment	Follow-Up (Range) (Months)	Mean Score Value before Surgery/Mean Score at Final Follow-Up (Used Score)	Return to Sports at the Same or Higher Level as before Surgery (%)
Kuwahata/1998 [60]	7 (N/A)	N/A	Herbert screw with bone peg transplantation	32 (NA)	N/A	100
Harada/2002 [87]	4 (4/0)	14.2 (14–15)	Staples with bone peg transplantation	90 (24–132)	N/A	75
Takeda/2002 [55]	11 (11/0)	14.7 (12–16)	K wires with bone peg transplantation	57 (31–95)	N/A	91
Nobuta/2008 [57]	28 (28/0)	14 (12–19)	Flexible wire or suture wire	17 (7–36)	N/A	86
Takeba/2010 [58]	4 (4/0)	14 (12–16)	Bioresorbable pins	6 (3–7)	N/A	N/A
Hennrikus/2015 [59]	26 (13/13)	14.1 (N/A)	Bioresorbable pins or K wires	39 (12–96)	70/100 (MEPS ^1^)	66
Koehler/2015 [88]	4 (1/3)	N/A (13–15)	Non-resorbable suture with bone autograft from the iliac crest	33 (31–36)	N/A/88 (MEPS ^1^) N/A/42 (OES ^2^)	100
Uchida/2015 [89]	18 (N/A)	14.2 (12–16)	Bioresorbable pins	39 (36–50)	127/197.5 (T&A ^3^) 68/98 (MEPS ^1^)	94
Takeba/2015 [90]	13 (N/A)	14 (12–16)	Bioresorbable pins	24 (12–50)	12.4/0.5 (DASH disability/symptom score ^4^) 74.5/1.4 (DASH sports score ^4^)	N/A
Kiyomatsu/2021 [91]	34 (N/A)	14 (12–16)	Bioresorbable pins	28.7 (24–50)	15.5/10.8(DASH disability/symptom score ^4^) 69.2/13.0 (DASH sports score ^4^)	82.3

^1^ MEPS—Mayo Elbow Performance score; ^2^ OES—Oxford Elbow score; ^3^ T&A—Timmerman and Andrews score; ^4^ Disabilities of Arm, Shoulder and Hand; N/A = unknown.

**Table 4:** Literature review of studies including general information about patients, surgical technique, and results after autologous osteochondral transplantation in patients with capitellar osteochondritis dissecans of the elbow.

First Author/Year of Publication	Number of Patients (Male/Female)	Mean Age (Range) at the Time of Surgery	Follow-Up (Range) (Months)	Mean Score Value before Surgery/Mean Score at Final Follow-Up (Used Score)	Return to Sports at the Same or Higher Level as before Surgery (%)
**Autologous Bone Transplantation from the Knee**					
Shimada/2005 [95]	10 (10/0)	14.3 (12–17)	26 (18–45)	80/94 (JOA ^1^)	80
Tsuda/2005 [92]	3 (2/1)	12.6 (12–13)	N/A	N/A	100
Yamamoto/2006 [96]	18 (18/0)	13.6 (10–16)	42 (25–63)	151/181 (T&A ^2^)	78
Iwasaki/ 2009 [97]	19 (19/0)	14.2 (11–19)	45 (24–87)	131/191 (T&A ^2^)	89
Nishimura/ 2011 [98]	12 (12/0)	14.4 (12–17)	34 (N/A)	N/A	N/A
Ovesen/ 2011 [99]	10 (4/6)	18.3 (13–27)	30 (10–60)	71/94 (MEPS ^3^)	N/A
Maruyama/2014 [100]	33 (33/0)	13.6 (11–17)	28 (12–76)	143/190 (T&A ^2^)	94
Weigelt/ 2015 [101]	14 (9/5)	18.3 (12–33)	85 (36–168)	N/A/95 (BMS ^4^)	62
Lyons/ 2015 [102]	11 (10/1)	14.5 (13–17)	23 (6–49)	N/A/1,4 (DASH ^5^)	100
Matsuura/2017 [103]	87 (86/1)	13.2 (11–16)	43.4 (24–100)	139.7/193.8 (T&A ^2^ for central lesions) 136.7/185.3 (T&A^2^ for lateral lesions)	100 (C); 86 (L)
Funakoshi/2018 [39]	22 (22/0)	13.5 (NA)	27.5 (24–48)	132.3/189.8 (T&A ^2^)	91
Wu/2018 [104]	31 (N/A)	N/A	N/A	N/A	100
Yamagami/2018 [105]	C 13 (N/A)	14 (12–17)	36 (16–56)	74.5/99.2 (JOA ^1^)	100
	L 9 (N/A)	14 (13–17)	45 (22–99)	63.7/95.4 (JOA ^1^)	100
Pederzini/2019 [106]	9 (7/2)	22.4 (17–45)	48 (30–52)	60/98.3 (MEPS ^3^) 44/2.5 (QuickDASH ^5^)	78
Bae/2020 [107]	28 (14/14)	14.2 (11.8–18.8)	6.3 (5–27) clinical 5.7 (5–26.7) radiological	150/190 (T&A ^2^)	69
Ueda/2021 [83]	29 (29/0)	14 (13–15)	84 (60–156)	126/175 (T&A ^2^) 48/1 (DASH ^5^)	90
**Autologous bone transplantation from the rib**					
Sato/2008 [108]	14 (14/0)	16.4 (13–25)	22 (6–56)	N/A	N/A
Mihara/2010 [52]	7 (7/0)	13.3 (11–15)	37.4 (24–92)	141/185 (T&A ^2^)	N/A
Shimada/ 2012 [94]	26 (26/0)	15.5 (12–43)	36 (24–51)	111/180 (T&A ^2^)	81 ^a^
Nishinaka/2014 [29]	22 (22/0)	13.9 (11–16)	27 (12–77)	122/169 (T&A ^2^) 53/86 (JOA ^1^)	82 ^b^
Sato/2018 [109]	72 (71/1)	14.3 (11–25)	57 (36–147)	122/169 (T&A ^2^)	97

^1^ Japanese Orthopaedic Association score; ^2^ Timmerman and Andrews score; ^3^ Mayo Elbow Performance Score; ^4^ Broberg–Morrey Score; ^5^ Disabilities of Arm, Shoulder, and Hand score; C = central OCD lesions; L = lateral localized OCD lesions; ^a^ five patients underwent revision surgery 18-24 months after the initial treatment and all of them returned to sports afterward; ^b^ four patients underwent revision surgery 18–68 months after the initial treatment and all of them returned to sports afterward; N/A = unknown.

## Data Availability

Data are contained within the article.

## References

[1] Watkins R.A., De Borja C., Ramirez F. (2022). Common Upper Extremity Injuries in Pediatric Athletes. Curr. Rev. Musculoskelet. Med..

[2] Maffulli N., Longo U.G., Gougoulias N., Caine D., Denaro V. (2011). Sport Injuries: A Review of Outcomes. Br. Med. Bull..

[3] Zaremski J.L., Zeppieri G., Tripp B.L. (2019). Injury Prevention Considerations in Adolescent Overhead-Throwing Athletes. Curr. Phys. Med. Rehabil. Rep..

[4] Pluim B.M. (2006). Tennis Injuries: Occurrence, Aetiology, and Prevention. Br. J. Sports Med..

[5] Cohen D., Kay J., Memon M., Slawaska-Eng D., Simunovic N., Ayeni O.R. (2021). A High Rate of Children and Adolescents Return to Sport after Surgical Treatment of Osteochondritis Dissecans of the Elbow: A Systematic Review and Meta-Analysis. Knee Surg. Sports Traumatol. Arthrosc..

[6] Smucny M., Kolmodin J., Saluan P. (2016). Shoulder and Elbow Injuries in the Adolescent Athlete. Sports Med. Arthrosc. Rev..

[7] Beck J.J., Richmond C.G., Tompkins M.A., Heyer A., Shea K.G., Cruz A.I. (2018). What’s New in Pediatric Upper Extremity Sports Injuries?. J. Pediatr. Orthop..

[8] Kohyama S., Ogawa T., Mamizuka N., Hara Y., Yamazaki M. (2018). A Magnetic Resonance Imaging–Based Staging System for Osteochondritis Dissecans of the Elbow: A Validation Study Against the International Cartilage Repair Society Classification. Orthop. J. Sports Med..

[9] Churchill R.W., Munoz J., Ahmad C.S. (2016). Osteochondritis Dissecans of the Elbow. Curr. Rev. Musculoskelet. Med..

[10] Logli A.L., Bernard C.D., O’Driscoll S.W., Sanchez-Sotelo J., Morrey M.E., Krych A.J., Camp C.L. (2019). Osteochondritis Dissecans Lesions of the Capitellum in Overhead Athletes: A Review of Current Evidence and Proposed Treatment Algorithm. Curr. Rev. Musculoskelet. Med..

[11] Hu H., Zhang C., Chen J., Li P., Zhang X., Deng Z., Du Y. (2019). Clinical Value of MRI in Assessing the Stability of Osteochondritis Dissecans Lesions: A Systematic Review and Meta-Analysis. AJR Am. J. Roentgenol..

[12] Logli A.L., Leland D.P., Bernard C.D., Sanchez-Sotelo J., Morrey M.E., O’Driscoll S.W., Krych A.J., Wang Z., Camp C.L. (2020). Capitellar Osteochondritis Dissecans Lesions of the Elbow: A Systematic Review of Osteochondral Graft Reconstruction Options. Arthroscopy.

[13] Dee D.T., Hung V.T., Schamblin C.J., Lupica G.M., Hitchens H.R., McGarry M.H., Lee T.Q. (2022). Radiocapitellar Contact Characteristics After Osteochondral Defect Repair Using a Novel Hybrid Reconstructive Procedure. Orthop. J. Sports Med..

[14] Powell G.M., Murthy N.S., Johnson A.C. (2021). Radiographic and MRI Assessment of the Thrower’s Elbow. Curr. Rev. Musculoskelet. Med..

[15] Kramer D.E. (2010). Elbow Pain and Injury in Young Athletes. J. Pediatr. Orthop..

[16] Patel H., Lala S., Helfner B., Wong T.T. (2021). Tennis Overuse Injuries in the Upper Extremity. Skeletal. Radiol..

[17] Ouellette H., Kassarjian A., Tétreault P., Palmer W. (2005). Imaging of the overhead throwing athlete. Semin. Musculoskelet. Radiol..

[18] Matsuura T., Suzue N., Kashiwaguchi S., Arisawa K., Yasui N. (2013). Elbow Injuries in Youth Baseball Players Without Prior Elbow Pain: A 1-Year Prospective Study. Orthop. J. Sports Med..

[19] Tisano B.K., Estes A.R. (2016). Overuse Injuries of the Pediatric and Adolescent Throwing Athlete. Med. Sci. Sports Exerc..

[20] Anderson M.W., Alford B.A. (2010). Overhead Throwing Injuries of the Shoulder and Elbow. Radiol. Clin. N. Am..

[21] Bruns J., Werner M., Habermann C.R. (2021). Osteochondritis Dissecans of Smaller Joints: The Elbow. Cartilage.

[22] Collins M.S., Tiegs-Heiden C.A. (2020). Osteochondral Lesions of the Lateral Trochlear Ridge: A Rare, Subtle but Important Finding on Advanced Imaging in Patients with Elbow Pain. Skeletal. Radiol..

[23] Wang K.K., Williams K., Bae D.S. (2020). Early Radiographic Healing and Functional Results After Autologous Osteochondral Grafting for Osteochondritis Dissecans of the Capitellum: Introduction of a New Magnetic Resonance Imaging–Based Scoring System. Am. J. Sports Med..

[24] Uvodich M.E., Braig Z.V., Reinholz A.K., Till S.E., O’Driscoll S.W., Morrey M.E., Sanchez-Sotelo J., Camp C.L. (2022). Incidence and Epidemiology of Symptomatic Capitellar Osteochondritis Dissecans of the Elbow: A United States Population–Based Study Over a 25-Year Period. Orthop. J. Sports Med..

[25] Takahara M., Uno T., Maruyama M., Harada M., Satake H., Takahara D., Takagi M. (2022). Staging of Osteochondritis Dissecans of the Elbow Based on Pathologic Progression in the Partially Detached Articular Fragment. J. Shoulder Elb. Surg..

[26] Michelin R.M., Gornick B.R., Schlechter J.A. (2022). Adolescent Athletes Achieve High Levels of Athletic and Daily Function After Arthroscopic Marrow Stimulation for Elbow Capitellar Osteochondritis Dissecans. Arthrosc. Sports Med. Rehabil..

[27] Wang K.K., Bixby S.D., Bae D.S. (2019). Osteochondritis Dissecans of the Humeral Trochlea: Characterization of a Rare Disorder Based on 28 Cases. Am. J. Sports Med..

[28] Micheloni G.M., Tarallo L., Negri A., Giorgini A., Merolla G., Porcellini G. (2021). Pediatric Elbow Arthroscopy: Clinical Outcomes and Complications after Long-Term Follow-Up. J. Orthop. Traumatol..

[29] Nishinaka N., Tsutsui H., Yamaguchi K., Uehara T., Nagai S., Atsumi T. (2014). Costal Osteochondral Autograft for Reconstruction of Advanced-Stage Osteochondritis Dissecans of the Capitellum. J. Shoulder Elb. Surg..

[30] Bizzoca D., Moretti L., Rifino F., Dibello D., Moretti B. (2021). Upper Limb Injures in Young Athletes. Minerva Orthop..

[31] Chauvin N.A., Gustas-French C.N. (2019). Magnetic Resonance Imaging of Elbow Injuries in Children. Pediatr. Radiol..

[32] Rotman D., Kwak J.-M., Rojas Lievano J., Hooke A., Camp C.L., Fitzsimmons J.S., O’Driscoll S.W. (2021). Radial Head Lag: A Possible Biomechanical Mechanism for Osteochondritis Dissecans of the Capitellum in Baseball Pitchers. Am. J. Sports Med..

[33] Gadinsky N.E., O’Brien M.J. (2020). Osteochondritis Dissecans of the Capitellum: Management in the Throwing Athlete. Oper. Tech. Sports Med..

[34] Wang Q. (2006). Baseball and Softball Injuries. Curr. Sports Med. Rep..

[35] Malik S.S., Rasodivic D., Saeed A., Jordan R.W., Maclean S., I Bain G. (2022). Management of Osteochondritis Dissecans (OCD) of the Elbow Trochlea in the Adolescent Population: A Systematic Review. Shoulder Elbow.

[36] Kamei K., Sasaki N., Sasaki E., Sasaki S., Kimura Y., Maeda S., Yamamoto Y., Ishibashi Y. (2021). Association Between Osteochondritis Dissecans of the Humeral Capitellum and Medial Epicondyle Lesion in Baseball Players. Orthop. J. Sports Med..

[37] Iwasaki N., Kamishima T., Kato H., Funakoshi T., Minami A. (2012). A Retrospective Evaluation of Magnetic Resonance Imaging Effectiveness on Capitellar Osteochondritis Dissecans Among Overhead Athletes. Am. J. Sports Med..

[38] Bexkens R., Oosterhoff J.H., Tsai T.-Y., Doornberg J.N., Van Den Bekerom M.P.J., Eygendaal D., Oh L.S. (2017). Osteochondritis Dissecans of the Capitellum: Lesion Size and Pattern Analysis Using Quantitative 3-Dimensional Computed Tomography and Mapping Technique. J. Shoulder Elb. Surg..

[39] Funakoshi T., Momma D., Matsui Y., Kamishima T., Matsui Y., Kawamura D., Nagano Y., Iwasaki N. (2018). Autologous Osteochondral Mosaicplasty for Centrally and Laterally Located, Advanced Capitellar Osteochondritis Dissecans in Teenage Athletes: Clinical Outcomes, Radiography, and Magnetic Resonance Imaging Findings. Am. J. Sports Med..

[40] Claessen F.M.A.P., Van Den Ende K.I.M., Doornberg J.N., Guitton T.G., Eygendaal D., Van Den Bekerom M.P.J., Van Der Lugt J., Schep N.W., Boerboom A.L., Van Der Pluim M. (2015). Osteochondritis Dissecans of the Humeral Capitellum: Reliability of Four Classification Systems Using Radiographs and Computed Tomography. J. Shoulder Elb. Surg..

[41] Pu A., Jauregui J.J., Salmons H.I., Weir T.B., Abzug J.M., Gilotra M.N. (2021). Radiographic Evaluation of Osteochondritis Dissecans of the Humeral Capitellum: A Systematic Review. J. Orthop..

[42] Matsui Y., Funakoshi T., Momma D., Miyamoto A., Endo K., Furushima K., Fujisaki K., Iwasaki N. (2018). Variation in Stress Distribution Patterns across the Radial Head Fovea in Osteochondritis Dissecans: Predictive Factors in Radiographic Findings. J. Shoulder Elb. Surg..

[43] Nissen C., Bohn D.C., Crepeau A., Edmonds E., Ganley T., Kostyun R., Lawrence J.T.R., Pace J.L., Saluan P., Uquillas C. (2022). Reliability of Radiographic Imaging Characteristics for Osteochondritis Dissecans of the Capitellum. Am. J. Sports Med..

[44] Kohyama S., Nishiura Y., Hara Y., Ogawa T., Ikumi A., Okano E., Totoki Y., Yoshii Y., Yamazaki M. (2021). Preoperative Evaluation and Surgical Simulation for Osteochondritis Dissecans of the Elbow Using Three-Dimensional MRI-CT Image Fusion Images. Diagnostics.

[45] Nguyen J.C., Degnan A.J., Barrera C.A., Hee T.P., Ganley T.J., Kijowski R. (2019). Osteochondritis Dissecans of the Elbow in Children: MRI Findings of Instability. AJR Am. J. Roentgenol..

[46] Yoshizuka M., Sunagawa T., Nakashima Y., Shinomiya R., Masuda T., Makitsubo M., Adachi N. (2018). Comparison of Sonography and MRI in the Evaluation of Stability of Capitellar Osteochondritis Dissecans. J. Clin. Ultrasound.

[47] Takahara M., Mura N., Sasaki J., Harada M., Ogino T. (2007). Classification, Treatment, and Outcome of Osteochondritis Dissecans of the Humeral Capitellum. J. Bone Jt. Surg. Am..

[48] Iwasaki N., Yamane S., Nishida K., Masuko T., Funakoshi T., Kamishima T., Minami A. (2010). Transplantation of Tissue-Engineered Cartilage for the Treatment of Osteochondritis Dissecans in the Elbow: Outcomes over a Four-Year Follow-up in Two Patients. J. Shoulder Elb. Surg..

[49] Dimnjaković D., Bojanić I., Mahnik A., Smoljanović T. (2013). Synovial Chondromatosis of the Elbow. Coll. Antropol..

[50] Bojanić I., Smoljanović T., Mahnik A. (2010). Arthroscopy of the elbow. Liječnički Vjesnik.

[51] Maruyama M., Harada M., Satake H., Tomohiro U., Takagi M., Takahara M. (2016). Bone-Peg Grafting for Osteochondritis Dissecans of the Humeral Capitellum. J. Orthop. Surg..

[52] Mihara K., Suzuki K., Makiuchi D., Nishinaka N., Yamaguchi K., Tsutsui H. (2010). Surgical Treatment for Osteochondritis Dissecans of the Humeral Capitellum. J. Shoulder Elb. Surg..

[53] Takahara M., Mura N., Sasaki J., Harada M., Ogino T. (2008). Classification, Treatment, and Outcome of Osteochondritis Dissecans of the Humeral Capitellum: Surgical Technique. J. Bone Jt. Surg. Am..

[54] Bojanić I., Smoljanović T., Dokuzović S. (2012). Osteochondritis Dissecans of the Elbow: Excellent Results in Teenage Athletes Treated by Arthroscopic Debridement and Microfracture. Croat. Med. J..

[55] Takeda H., Watarai K., Matsushita T., Saito T., Terashima Y. (2002). A Surgical Treatment for Unstable Osteochondritis Dissecans Lesions of the Humeral Capitellum in Adolescent Baseball Players. Am. J. Sports Med..

[56] Nobuta S., Sato K., Kasama F., Hatori M., Itoi E. (2008). Open Elbow Arthrolysis for Post-Traumatic Elbow Contracture. Upsala J. Med. Sci..

[57] Nobuta S., Ogawa K., Sato K., Nakagawa T., Hatori M., Itoi E. (2008). Clinical Outcome of Fragment Fixation for Osteochondritis Dissecans of the Elbow. Upsala J. Med. Sci..

[58] Takeba J., Takahashi T., Hino K., Watanabe S., Imai H., Yamamoto H. (2010). Arthroscopic Technique for Fragment Fixation Using Absorbable Pins for Osteochondritis Dissecans of the Humeral Capitellum: A Report of 4 Cases. Knee Surg. Sports Traumatol. Arthrosc..

[59] Hennrikus W.P., Miller P.E., Micheli L.J., Waters P.M., Bae D.S. (2015). Internal Fixation of Unstable In Situ Osteochondritis Dissecans Lesions of the Capitellum. J. Pediatr. Orthop..

[60] Kuwahata Y., Inoue G. (1998). Osteochondritis Dissecans of the Elbow Managed by Herbert Screw Fixation. Orthopedics.

[61] Baumgarten T.E., Andrews J.R., Satterwhite Y.E. (1998). The Arthroscopic Classification and Treatment of Osteochondritis Dissecans of the Capitellum. Am. J. Sports Med..

[62] Bojanić I., Ivković A., Borić I. (2006). Arthroscopy and Microfracture Technique in the Treatment of Osteochondritis Dissecans of the Humeral Capitellum: Report of Three Adolescent Gymnasts. Knee Surg. Sports Traumatol. Arthrosc..

[63] McLaughlin R.J., Leland D.P., Bernard C.D., Sanchez-Sotelo J., Morrey M.E., O’Driscoll S.W., Camp C.L. (2021). Both Debridement and Microfracture Produce Excellent Results for Osteochondritis Dissecans Lesions of the Capitellum: A Systematic Review. Arthrosc. Sports Med. Rehabil..

[64] Patel S.P., Conyer R.T., Shybut T.B. (2020). Osteochondral Autograft Transfer for Capitellar Chondral and Osteochondral Defects. Arthrosc. Tech..

[65] Kolmodin J., Saluan P. (2014). Osteochondritis Dissecans of the Humeral Capitellum: The Significance of Lesion Location. Orthop. J. Sports Med..

[66] Mihata T., Quigley R., Robicheaux G., McGarry M.H., Neo M., Lee T.Q. (2013). Biomechanical Characteristics of Osteochondral Defects of the Humeral Capitellum. Am. J. Sports Med..

[67] Shi L.L., Bae D.S., Kocher M.S., Micheli L.J., Waters P.M. (2012). Contained versus Uncontained Lesions in Juvenile Elbow Osteochondritis Dissecans. J. Pediatr. Orthop..

[68] Bexkens R., Ogink P.T., Doornberg J.N., Kerkhoffs G.M.M.J., Eygendaal D., Oh L.S., van den Bekerom M.P.J. (2017). Donor-Site Morbidity after Osteochondral Autologous Transplantation for Osteochondritis Dissecans of the Capitellum: A Systematic Review and Meta-Analysis. Knee Surg. Sports Traumatol. Arthrosc..

[69] Funakoshi T., Furushima K., Miyamoto A., Kusano H., Horiuchi Y., Itoh Y. (2019). Predictors of Unsuccessful Nonoperative Management of Capitellar Osteochondritis Dissecans. Am. J. Sports Med..

[70] Obey M.R., Hillen T.J., Broughton J.S., Smith M.V., Goldfarb C.A. (2022). Magnetic Resonance Imaging Assessment of Cartilage Appearance Following Marrow Stimulation of Osteochondritis Dissecans of the Humeral Capitellum. J. Hand Surg. Am..

[71] Ruch D.S., Cory J.W., Poehling G.G. (1998). The Arthroscopic Management of Osteochondritis Dissecans of the Adolescent Elbow. Arthroscopy.

[72] Byrd J.W.T., Jones K.S. (2002). Arthroscopic Surgery for Isolated Capitellar Osteochondritis Dissecans in Adolescent Baseball Players: Minimum Three-Year Follow-Up. Am. J. Sports Med..

[73] Brownlow H.C., O’Connor-Read L.M., Perko M. (2006). Arthroscopic Treatment of Osteochondritis Dissecans of the Capitellum. Knee Surg. Sports Traumatol. Arthrosc..

[74] Rahusen F.T.G., Brinkman J.M., Eygendaal D. (2006). Results of Arthroscopic Debridement for Osteochondritis Dissecans of the Elbow. Br. J. Sports Med..

[75] Schoch B., Wolf B.R. (2010). Osteochondritis Dissecans of the Capitellum: Minimum 1-Year Follow-up after Arthroscopic Debridement. Arthroscopy.

[76] Miyake J., Masatomi T. (2011). Arthroscopic Debridement of the Humeral Capitellum for Osteochondritis Dissecans: Radiographic and Clinical Outcomes. J. Hand Surg. Am..

[77] Tis J.E., Edmonds E.W., Bastrom T., Chambers H.G. (2012). Short-Term Results of Arthroscopic Treatment of Osteochondritis Dissecans in Skeletally Immature Patients. J. Pediatr. Orthop..

[78] Wulf C.A., Stone R.M., Giveans M.R., Lervick G.N. (2012). Magnetic Resonance Imaging after Arthroscopic Microfracture of Capitellar Osteochondritis Dissecans. Am. J. Sports Med..

[79] Lewine E.B., Miller P.E., Micheli L.J., Waters P.M., Bae D.S. (2016). Early Results of Drilling and/or Microfracture for Grade IV Osteochondritis Dissecans of the Capitellum. J. Pediatr. Orthop..

[80] Bexkens R., van den Ende K.I.M., Ogink P.T., van Bergen C.J.A., van den Bekerom M.P.J., Eygendaal D. (2017). Clinical Outcome After Arthroscopic Debridement and Microfracture for Osteochondritis Dissecans of the Capitellum. Am. J. Sports Med..

[81] Ueda Y., Sugaya H., Takahashi N., Matsuki K., Tokai M., Onishi K., Hoshika S., Hamada H. (2017). Arthroscopic Fragment Resection for Capitellar Osteochondritis Dissecans in Adolescent Athletes: 5- to 12-Year Follow-Up. Orthop. J. Sports Med..

[82] Matsuura T., Iwame T., Suzue N., Kashiwaguchi S., Iwase T., Hamada D., Sairyo K. (2020). Long-Term Outcomes of Arthroscopic Debridement With or Without Drilling for Osteochondritis Dissecans of the Capitellum in Adolescent Baseball Players: A ≥10-Year Follow-Up Study. Arthroscopy.

[83] Ueda Y., Sugaya H., Takahashi N., Matsuki K., Tokai M., Morioka T., Hoshika S., Takeuchi Y. (2021). Comparison Between Osteochondral Autograft Transplantation and Arthroscopic Fragment Resection for Large Capitellar Osteochondritis Dissecans in Adolescent Athletes: A Minimum 5 Years’ Follow-Up. Am. J. Sports Med..

[84] Austin D.C., Song B., Rojas Lievano J.L., Rogers T.H., Barlow J.D., Camp C.L., Morrey M.E., Sanchez-Sotelo J.L., Fitzsimmons J.S., O’Driscoll S.W. (2023). Long-Term Patient-Reported Outcomes After Arthroscopic Debridement of Grade 3 or 4 Capitellar Osteochondritis Dissecans Lesions. Am. J. Sports Med..

[85] Rothermich M.A., Mussell E.A., Ryan M.K., Fleisig G.S., Sitton S.E., Emblom B.A., Dugas J.R., Andrews J.R., Cain E.L. (2023). Clinical Outcomes of Osteochondritis Dissecans Lesions of the Capitellum Treated with Arthroscopy with a Mean Follow-up Period of 8.3 Years. J. Shoulder Elb. Surg..

[86] Momma D., Onodera T., Kawamura D., Urita A., Matsui Y., Baba R., Funakoshi T., Kondo M., Endo T., Kondo E. (2021). Acellular Cartilage Repair Technique Based on Ultrapurified Alginate Gel Implantation for Advanced Capitellar Osteochondritis Dissecans. Orthop. J. Sports Med..

[87] Harada M., Ogino T., Takahara M., Ishigaki D., Kashiwa H., Kanauchi Y. (2002). Fragment Fixation with a Bone Graft and Dynamic Staples for Osteochondritis Dissecans of the Humeral Capitellum. J. Shoulder Elb. Surg..

[88] Koehler S.M., Walsh A., Lovy A.J., Pruzansky J.S., Shukla D.R., Hausman M.R. (2015). Outcomes of Arthroscopic Treatment of Osteochondritis Dissecans of the Capitellum and Description of the Technique. J. Shoulder Elb. Surg..

[89] Uchida S., Utsunomiya H., Taketa T., Sakoda S., Hatakeyama A., Nakamura T., Sakai A. (2015). Arthroscopic Fragment Fixation Using Hydroxyapatite/Poly-L-Lactate Acid Thread Pins for Treating Elbow Osteochondritis Dissecans. Am. J. Sports Med..

[90] Takeba J., Takahashi T., Watanabe S., Imai H., Kikuchi S., Umakoshi K., Matsumoto H., Ohshita M., Miura H., Aibiki M. (2015). Short-Term Clinical Results of Arthroscopic Osteochondral Fixation for Elbow Osteochondritis Dissecans in Teenaged Baseball Players. J. Shoulder Elb. Surg..

[91] Kiyomatsu H., Takeba J., Imai H., Fujibuchi T., Inoue T., Jono A., Hino K., Miura H. (2021). Treatment of Osteochondritis Dissecans of the Humeral Capitellum with a Fragment Fixation Method Using Absorbable Pins. JSES Int..

[92] Tsuda E., Ishibashi Y., Sato H., Yamamoto Y., Toh S. (2005). Osteochondral Autograft Transplantation for Osteochondritis Dissecans of the Capitellum in Nonthrowing Athletes. Arthroscopy.

[93] Smith M.V., Bedi A., Chen N.C. (2012). Surgical Treatment for Osteochondritis Dissecans of the Capitellum. Sports Health.

[94] Shimada K., Tanaka H., Matsumoto T., Miyake J., Higuchi H., Gamo K., Fuji T. (2012). Cylindrical Costal Osteochondral Autograft for Reconstruction of Large Defects of the Capitellum Due to Osteochondritis Dissecans. J. Bone Jt. Surg. Am..

[95] Shimada K., Yoshida T., Nakata K., Hamada M., Akita S. (2005). Reconstruction with an Osteochondral Autograft for Advanced Osteochondritis Dissecans of the Elbow. Clin. Orthop. Relat. Res..

[96] Yamamoto Y., Ishibashi Y., Tsuda E., Sato H., Toh S. (2006). Osteochondral Autograft Transplantation for Osteochondritis Dissecans of the Elbow in Juvenile Baseball Players: Minimum 2-Year Follow-Up. Am. J. Sports Med..

[97] Iwasaki N., Kato H., Ishikawa J., Masuko T., Funakoshi T., Minami A. (2009). Autologous Osteochondral Mosaicplasty for Osteochondritis Dissecans of the Elbow in Teenage Athletes. J. Bone Jt. Surg. Am..

[98] Nishimura A., Morita A., Fukuda A., Kato K., Sudo A. (2011). Functional Recovery of the Donor Knee after Autologous Osteochondral Transplantation for Capitellar Osteochondritis Dissecans. Am. J. Sports Med..

[99] Ovesen J., Olsen B.S., Johannsen H.V. (2011). The Clinical Outcomes of Mosaicplasty in the Treatment of Osteochondritis Dissecans of the Distal Humeral Capitellum of Young Athletes. J. Shoulder Elb. Surg..

[100] Maruyama M., Takahara M., Harada M., Satake H., Takagi M. (2014). Outcomes of an Open Autologous Osteochondral Plug Graft for Capitellar Osteochondritis Dissecans: Time to Return to Sports. Am. J. Sports Med..

[101] Weigelt L., Siebenlist S., Hensler D., Imhoff A.B., Vogt S. (2015). Treatment of Osteochondral Lesions in the Elbow: Results after Autologous Osteochondral Transplantation. Arch. Orthop. Trauma Surg..

[102] Lyons M.L., Werner B.C., Gluck J.S., Freilich A.M., Dacus A.R., Diduch D.R., Chhabra A.B. (2015). Osteochondral Autograft Plug Transfer for Treatment of Osteochondritis Dissecans of the Capitellum in Adolescent Athletes. J. Shoulder Elb. Surg..

[103] Matsuura T., Hashimoto Y., Nishino K., Nishida Y., Takahashi S., Shimada N. (2017). Comparison of Clinical and Radiographic Outcomes Between Central and Lateral Lesions After Osteochondral Autograft Transplantation for Osteochondritis Dissecans of the Humeral Capitellum. Am. J. Sports Med..

[104] Wu M., Eisenberg K., Williams K., Bae D.S. (2018). Radial Head Changes in Osteochondritis Dissecans of the Humeral Capitellum. Orthop. J. Sports Med..

[105] Yamagami N., Yamamoto S., Aoki A., Ito S., Uchio Y. (2018). Outcomes of Surgical Treatment for Osteochondritis Dissecans of the Elbow: Evaluation by Lesion Location. J. Shoulder Elb. Surg..

[106] Pederzini L.A., Bartoli M., Cheli A., Nicoletta F., Severini G. (2019). Encouraging Mid-Term Outcomes for Arthroscopic Autologous Osteochondral Transplant (OAT) in Capitellum Osteochondritis Dissecans (OCD). Knee Surg. Sports Traumatol. Arthrosc..

[107] Bae D.S., Ingall E.M., Miller P.E., Eisenberg K. (2020). Early Results of Single-Plug Autologous Osteochondral Grafting for Osteochondritis Dissecans of the Capitellum in Adolescents. J. Pediatr. Orthop..

[108] Sato K., Nakamura T., Toyama Y., Ikegami H. (2008). Costal Osteochondral Grafts for Osteochondritis Dissecans of the Capitulum Humeri. Tech. Hand Up. Extremity Surg..

[109] Sato K., Iwamoto T., Matsumura N., Suzuki T., Nishiwaki Y., Oka Y., Nakamura T. (2018). Costal Osteochondral Autograft for Advanced Osteochondritis Dissecans of the Humeral Capitellum in Adolescent and Young Adult Athletes: Clinical Outcomes with a Mean Follow-up of 4.8 Years. J. Bone Jt. Surg. Am..

[110] Sato M., Ochi M., Uchio Y., Agung M., Baba H. (2004). Transplantation of Tissue-Engineered Cartilage for Excessive Osteochondritis Dissecans of the Elbow. J. Shoulder Elb. Surg..

[111] Kircher J. (2016). Autologous Chondrocyte Implantation for Post-Traumatic Cartilage Defect of the Capitulum Humeri. J. Shoulder Elb. Surg..

[112] Patzer T., Krauspe R., Hufeland M. (2016). Arthroscopic Autologous Chondrocyte Transplantation for Osteochondritis Dissecans of the Elbow. Arthrosc. Tech..

[113] Chawla S., Saper M.G. (2020). Fresh Precut Osteochondral Allograft Core Transplantation for the Treatment of Capitellum Osteochondritis Dissecans. Arthrosc. Tech..

[114] Mirzayan R., Lim M.J. (2016). Fresh Osteochondral Allograft Transplantation for Osteochondritis Dissecans of the Capitellum in Baseball Players. J. Shoulder Elb. Surg..

[115] Goldstein Z.T., Thompson A.R., Robbins M.A., Yang S.S., Nazir O.F., Mirarchi A.J. (2021). Optimizing Graft Extraction From the Femoral Condyle for Fresh Osteochondral Allograft Transplantation in Treating Osteochondritis Dissecans of the Capitellum: Best Fit Based on Radius of Curvature. J. Pediatr. Orthop..

[116] Wilk K.E., Macrina L.C., Cain E.L., Dugas J.R., Andrews J.R. (2012). Rehabilitation of the Overhead Athlete’s Elbow. Sports Health.

